# Serum and exosome WNT5A levels as biomarkers in non-small cell lung cancer

**DOI:** 10.1186/s12931-025-03216-7

**Published:** 2025-04-13

**Authors:** Zsofia Torok, Kitti Garai, Judit Bovari-Biri, Zoltan Adam, Judith A Miskei, Bela Kajtar, Veronika Sarosi, Judit E Pongracz

**Affiliations:** 1 Department of Pharmaceutical Biotechnology, Faculty of Pharmacy, University of Pecs, 2 Rokus Str, Pecs, Pecs, H-7624 Hungary; 2 Department of Pulmonology, 1st Internal Medicine, The Medical School and Clinical Centre, University of Pecs, 12 Szigeti Str, Pecs, H-7624 Hungary; 3 Department of Pathology, The Medical School and Clinical Centre, University of Pecs, 12 Szigeti Str, Pecs, H-7624 Hungary

**Keywords:** WNT5A, Exosome, Non-small cell lung cancer, Lung squamous cell carcinoma, Lung adenocarcinoma

## Abstract

**Background:**

Despite significant advances in the treatment of lung cancer (LC), there are no reliable biomarkers to effectively predict therapy response and overall survival (O/S) in non-small cell lung cancer (NSCLC) subtypes. While targeted therapies have improved survival rates in lung adenocarcinoma (LUAD), effective treatment options for lung squamous cell carcinoma (LUSC) are still limited. Recent evidence indicates that exosome-bound WNT5A may significantly contribute to disease progression. Our study assessed the WNT5A protein as a potential biomarker for diagnosing patients and predicting prognosis to assist in therapy selection.

**Methods:**

Primary tumor tissue and serum samples were collected from a cohort of 60 patients with histologically confirmed NSCLC before therapy. Healthy serum donors served as controls. Exosomes were isolated, then exosome number and size were measured, and WNT5A protein levels were identified in tissue and in vesicle-free, vesicle-bound fractions of the serum by ELISA.

**Results:**

Extensive statistical analysis (ROC, AUC, Cox, etc.) revealed that elevated WNT5A levels on the serum-exosome surface correlated with distant metastasis, advanced disease stage, and lymph node involvement in LUSC but not in LUAD patients. Moreover, a high WNT5A exosome surface expression was associated with a poor response to therapy and shorter O/S in LUSC patients. Additionally, serum-exosome surface + cargo WNT5A content distinguished LUAD and LUSC subtypes.

**Conclusions:**

WNT5A, particularly its serum exosome-bound form, may serve as a valuable biomarker after further validation for differentiating NSCLC subtypes and predicting disease progression. Importantly, the information can become available from a simple serum sample at the time of diagnosis.

**Supplementary Information:**

The online version contains supplementary material available at 10.1186/s12931-025-03216-7.

## Background

By 2020, LC became the second most common cancer and the leading cause of death worldwide [1, 2]. NSCLC represents 85% of all LC cases, with LUAD comprising 50% and LUSC accounting for 30% [3]. Recent reports by the American Society of Clinical Oncology (ASCO) have shown significant progress in treating NSCLC [4]. Nonetheless, the 5-year survival of LC is still lower than any other cancer type [5]. Many LUAD subtypes have well-described mutations that can be targeted with specific drugs that increase survival. The most well-known mutations that determine therapy in LUAD patients include KRAS, EGFR, and ALK mutations [6]. In addition to the traditionally tested therapy targets, next-generation sequencing (NGS) identified mutations in *BRAF*,* MET*,* ROS1*,* RET*,* NTRK1/2/3*, and *ERBB2* that offer additional therapy targets [7]. In contrast to LUAD, LUSC is more strongly associated with cigarette smoking, resulting in a highly variable mutational background, often in combinations of gene amplifications (*CCND1/2/3*,* CDK4*,* FGFR1/2/3*,* MET*,* PDGFRA*,* PIK3CA*,* SOX2*), gene fusions (*FGFR3::TACC3*), tumour suppressor mutations (*PTEN*,* TP53*) and point mutations (*EPHA2*,* AKT1*,* DDR2*) in a variety of genes [8]. Therefore, to date, mutation-based targeted therapy is not available for LUSC patients. Although immune checkpoint inhibitors are used to treat patients with LUAD and LUSC, the therapeutic response remains variable [9].

The answer potentially lies in the complexity of the carcinogenic process, where not just genetic mutations are responsible for the disease outcome but highly diverse and malfunctioning signalling pathways, including the WNT pathways [10]. In the carcinogenic process, the transforming members of the WNT ligand family have been investigated in depth [11]. One of the non-transforming family members, WNT5A, has been identified in several studies as one of the primary regulators of a wide range of squamous cell carcinomas (SCCs), including oral SCC [12], oesophagal SCC [13], cutaneous SCC [14], and lung SCC (LUSC) [11, 15–17]. Although WNT5A lacks transforming activity, it occasionally signals via the canonical or β-catenin-dependent pathway, which is the traditional signalling pathway for transforming canonical WNT ligands [10]. The secreted WNT5A is primarily known as a noncanonical WNT ligand that contributes less to the initiation of carcinogenesis but strongly supports cancer progression [18] by affecting cell migration, invasion, inflammation, and angiogenesis by diversely binding to the Frizzled (FZD), RYK, and ROR2 receptors [19]. Comparative analysis of WNT pathways in primary LUAD and LUSC tumour tissues revealed increased mRNA levels of the noncanonical WNT5A in LUSC [20, 21]. In a recent clinical study in which the expression and clinical impact of the WNT5A, WNT7B, FZD7 and GPC1 proteins were investigated in tumour samples, LUSC patients with no or minimal increase in WNT5A protein levels had the longest survival [20].

There are still discrepancies regarding the role of WNT5A in the process of carcinogenesis. As most of the studies focused on WNT5A protein at the tumour tissue level, less information is available on circulating ligands and their effects. To address where WNT5A is located, WNT5A in tumour tissues were compared with extracellular, circulating WNT5A in patient sera, both as vesicle-bound and vesicle-free soluble molecules. The lipophile [22] WNT5A can be transported by several lipid-containing carrier structures, including the secreted WNT interacting protein, heparan sulphate proteoglycans (HSPGs), cytonemes and the most broadly studied members of the extracellular vesicles (EV), exosomes [23]. The prognostic, diagnostic or predictive role of serum exosome-bound and exosome-free soluble WNT5A in LUAD and LUSC patients has not been investigated yet. Building on previous research that revealed varying concentrations of WNT5A in LUAD and LUSC in tumour tissue the present study sought to investigate WNT5A levels bound to exosomes and in blood serum samples devoid of EVs including exosomes, to reveal the role of WNT5A as a potential biomarker for clinical applications including disease progression, therapy response and patient survival [16, 24].

## Materials and methods

### Database analysis

Independent data set was analysed for WNT5A protein from using the Human Protein Atlas CPTACdataset, to support protein levels of WNT5A in LUAD and LUSC tumor tissues for validation of tissue WNT5A levels in the study cohort [24, 25].

### Patient and serum

The study was designed following the REMARK (REporting recommendations for tumour MARKer prognostic studies) criteria for tumour marker studies [26].

Sixty patients enrolled in the prospective study at the Department of Pulmonology, 1st Division Internal Medicine, Clinical Centre, University of Pecs (Pecs, Hungary) between 01.02.2018 and 15.12.2018. Each participant signed an informed consent form and blood serum samples were collected before any treatment began. After the diagnosis of pulmonary carcinoma, rule-based therapy and data collection followed. Where surgery was possible, tissue samples were also collected and stored at the Department of Pathology, Clinical Centre, University of Pecs (Pecs, Hungary). The inclusion criteria were as follows: (i) histologically confirmed NSCLC stage I-IV (LUAD or LUSC) based on American Joint Committee on Cancer (AJCC, version 8) staging; (ii) no history of radiotherapy, immunotherapy, chemotherapy, or other treatments before diagnosis; (iii) availability of complete follow-up data including best overall response (BOR) and overall survival (OS); and (iv) availability of sufficient quantity and quality of serum samples. The exclusion criteria were as follows: (i) pathologically different diagnoses of NSCLC; (ii) patients with a history of a second primary malignancy; and (iii) serum samples or any other reason for the failure of quality control at any stage of the study. BOR was determined according to RECIST version 1.1 [27]. The time from the start of treatment to the end of any cause or the last follow-up date was defined as OS. Serum from healthy blood donors (HC) selected by a comparable age distribution to the study population were used as controls. The study was declared complete three years later, in 2021. The study was approved by the local Research Ethics Committee, University of Pecs (PTE_KK_RIKEB_6444/2016) and was conducted in compliance with the Declaration of Helsinki.

### Immunohistochemistry

Immunohistochemistry was performed on 4 μm thick tissue sections of FFPE tissue blocks. Tris/EDTA buffer, pH 9.0, was used for 20 min for antigen retrieval. Reactions were visualised via BOND polymer refine detection (Leica DS9800) (Leica Biosystems, Deer Park, IL, United States). A monoclonal antibody WNT5A (Clone 3D10) (MA5-15511, Thermo Fisher Scientific, Waltham, MA, United States) was used.

### Extraction of exosomes from serum

Blood samples were collected, allowed to clot at 37 °C for 20 min, and then centrifuged at 1500×g for 10 min at room temperature (RT). The serum was stored at -80 °C until further processing. Exosomes were isolated from 400 µl of serum samples using Total Exosome Isolation Reagent (TEI) (from serum) (4478360, Invitrogen, Thermo-Fisher Scientific, Waltham, MA, United States). Briefly, serum samples were spun at 2000× g for 30 min to remove cells and debris. Next, 0.2 volumes of TEI reagent were added to each supernatant, and the samples were incubated at 4 °C for 30 min. The precipitated exosomes were recovered by centrifugation at 10,000×g for 10 min at RT. The exosome pellets were subsequently resuspended in PBS, pH 7.4, at RT.

### Nanoparticle tracking analysis

A NanoSight NS300 instrument (Malvern Panalytical Ltd., Malvern, United Kingdom) equipped with a 488-nm blue laser was used for real-time tracking and analysis.

All analysed samples were diluted in Ca- and Mg-free PBS to a final volume of 1 ml. Exosome isolates were subsequently diluted to the optimum NTA detection range before measurements (10–50 particles/frame). For each measurement, five 1-minute videos were captured under the following conditions: cell temperature: 25 °C; syringe speed: 50 µl/s. The videos were analysed using the in-built NTA v3.2 software.

### Transmission electron microscopy (TEM)

TEM was used to visualise the exosomes. A 2.5 µl sample volume was placed individually on a 300-mesh grid of each sample. The grid was dried overnight at RT and then 5% uranyl acetate and 3% sodium citrate were added to the grid. After 5 min of incubation, the grid was air-dried. Twenty-four hours later, the grid was analysed using JEOL TEM (JEOL Ltd., Tokyo, Japan) 1,200 EX.

### EV antibody array

EV-specific marker analysis was performed using the Exo-Check antibody array (EXORAY210B-8, System Biosciences, Palo Alto, CA, USA). 60 µg of EV preparation was added to the membrane-based blot array, and the manufacturer’s instructions were followed. The intensity of chemiluminescence was detected with a G: BOX Chemi XRQ (Syngene, Cambridge, UK).

### WNT5A ELISA

A human WNT5A (protein Wnt-5a) ELISA Kit (EH1164, Fine-Test, Wuhan, China) was used to quantify the serum, exosome-free serum, and exosome WNT5A contents. The starting volume was 400 µl of serum in all cases. To study the association and distribution of WNT5A with exosomes, the isolated pellet was resuspended in 495 µl of PBS to quantify the WNT5A content on the surface of exosomes. To determine the total WNT5A content of exosomes, the samples were resuspended in 400 µl PBS, then treated with 95 µl of ice-cold RIPA buffer (89900, Pierce RIPA buffer, Thermo-Fisher Scientific, Waltham, MA, United States) to disrupt the exosome membranes. All samples were incubated with 5 µl of 100x Halt-Protease inhibitor cocktail (87786, Thermo-Fisher Scientific, Waltham, MA, United States). The samples were mixed with their respective buffers and incubated on ice for 15 min. The protein concentration was quantified by a BCA assay using the Pierce™ BCA Protein Assay Kit (23225, Thermo-Fisher Scientific, Waltham, MA, United States) according to the manufacturer’s instructions.

All reagents, standards, and samples were prepared according to the manufacturer’s instructions. The absorbance of the samples was recorded at 450 nm via a Perkin Elmer Enspire Multiplate-Reader (Perkin Elmer, Waltham, MA, USA). The target concentrations were interpolated from a standard curve created from the standards with predefined concentrations. Each sample was measured in duplicate. The intra-assay variation coefficient was 4.1%, whereas the inter-assay variation coefficient was 14%. Evaluation of the ELISA was conducted without the knowledge of the clinical data to ensure objectivity.

### WNT5A protein per particle

The amount of WNT5A protein/particle was calculated as follows:

The amount of WNT5A protein/particle= $\:\frac{\text{W}\text{N}\text{T}5\text{A}\:\text{c}\text{o}\text{n}\text{c}\text{e}\text{n}\text{t}\text{r}\text{a}\text{t}\text{i}\text{o}\text{n}\:\text{m}\text{e}\text{a}\text{s}\text{u}\text{r}\text{e}\text{d}\:\text{b}\text{y}\:\text{E}\text{L}\text{I}\text{S}\text{A}\:(\text{p}\text{g}/\text{m}\text{L})}{\text{P}\text{a}\text{r}\text{t}\text{i}\text{c}\text{l}\text{e}\:\text{c}\text{o}\text{n}\text{c}\text{e}\text{n}\text{t}\text{r}\text{a}\text{t}\text{i}\text{o}\text{n}\:\text{d}\text{e}\text{t}\text{e}\text{c}\text{t}\text{e}\text{d}\:\text{b}\text{y}\:\text{N}\text{T}\text{A}\:(\text{p}\text{a}\text{r}\text{t}\text{i}\text{c}\text{l}\text{e}\text{s}/\text{m}\text{L})}$

### Statistical analysis

Data are expressed as the means ± SDs. The normal distribution was tested by Shapiro-Wilk test. The Mann‒Whitney U test, the Fisher’s exact test, and the unpaired t-test were used for the comparison of two specifications. The Kruskal-Wallis test, one-way ANOVA and the chi-squared test was used for comparison of three or more specifications. Cut-off values for serum, exosome-free serum, and exosome WNT5A were determined by receiver operating characteristics (ROC) curve analysis or the best cut-off method, a web-based survival analysis tool tailored for medical research (KMplot) [28]. Briefly, all possible cut-off values between the lower and upper quartiles are computed, and the best-performing threshold is used as a cut-off. The Kaplan‒Meier method was used to generate survival curves based on the length of time between primary treatment and exit. The log-rank test (Mantel‒Cox) was used to compare the survival distributions. Multivariable analysis was carried out by the Cox proportional hazards model. For all analyses, a two-sided *p* < 0.05 was considered statistically significant. Statistical analysis was performed using GraphPad Prism 9.0.0 software (GraphPad, Palo Alto, USA) or R (The R Foundation for Statistical Computing, Vienna, Austria).

## Results

### Patient characteristics

**Fig. 1:**
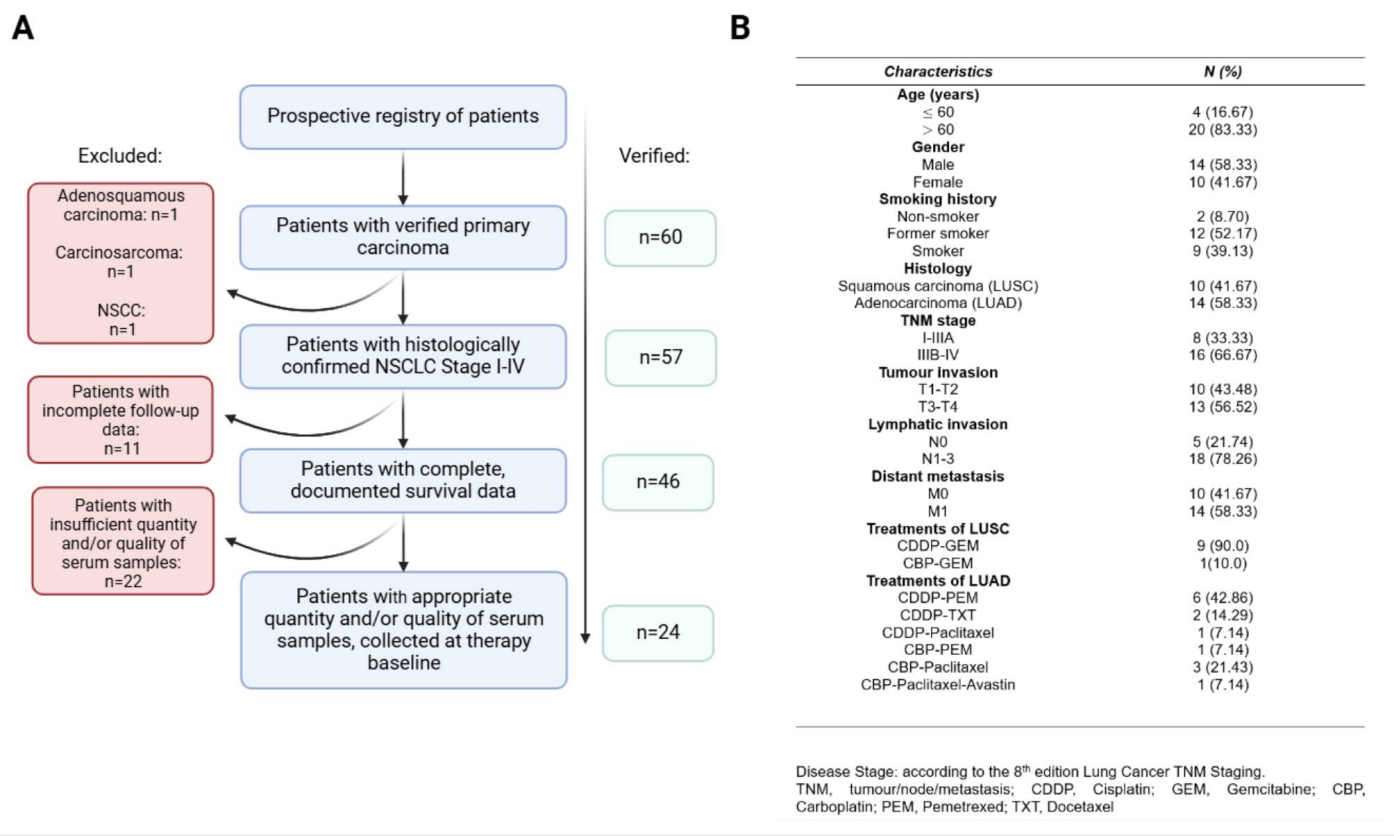
Patient selection and clinical parameters. (**A**) Flow chart of patient selection in our prospective study; **(B)** Clinicopathologic parameters of NSCLC patients

Out of the 60 patients who had verified primary lung carcinoma, 46 patients had complete follow-up data over the three-year study. Out of the 46 patients, only 24 (52.2%) patients had suitable quality and quantity of pretreatment serum samples. The detailed study flow according to REMARK criteria is described in Fig. 1A-B summarises patient baseline characteristics and the eventually applied therapy [29].

Out of the 24 NSCLC patients, the LUAD and LUSC subtypes represented 58.3% and 41.7%, respectively. Eight (33.3%) of the 24 patients were diagnosed with stage I-IIIA NSCLC, and 16 (66.67%) were at stage IIIB-IV. Additionally, most of the patients presented positive nodal status (78.3%) and had distant metastasis (58.3%). Based on the initial clinical parameters, Fisher’s exact and chi-squared tests were performed, and the two subtypes were comparable for baseline characteristics (Supplementary Table 1).

BOR was evaluated in 22 patients. From 14 LUAD patients, 4 (28.6%) showed an objective response [complete response (CR) + partial response (PR)]. SD was the best therapeutic outcome in 6 patients (42.9%), and 4 patients (28.6%) had PD. LUAD patients exited after a median survival time of 579 days. By contrast, of 10 LUSC patients, none of them had an objective therapy response, while 5 patients (50%) had SD, and 3 patients (30%) had PD. LUSC patients exited after a median survival time of 280 days.

### WNT5A protein in the tumour tissue

In our operable patient cohort, we did not detect significant differences in WNT5A protein levels in LUAD and LUSC tumour tissues (Supplementary Fig. 1A-B-). Analysis of independent protein CPTAC datasets supported these results (Supplementary Fig. 1C). Additionally, our Kaplan-Meier analysis revealed no correlation between tumour tissue WNT5A levels and OS (logrank *p* = 0.14, HR = 0.41, 95% CI 0.12–1.39) (Supplementary Fig. 1D). In line with previous research, our study demonstrated that LUAD patients survived significantly longer than LUSC patients after diagnosis (*p* = 0.0193) (Supplementary Fig. 1-D and E).

### Characterisation of exosomes extracted from serum

**Fig. 2:**
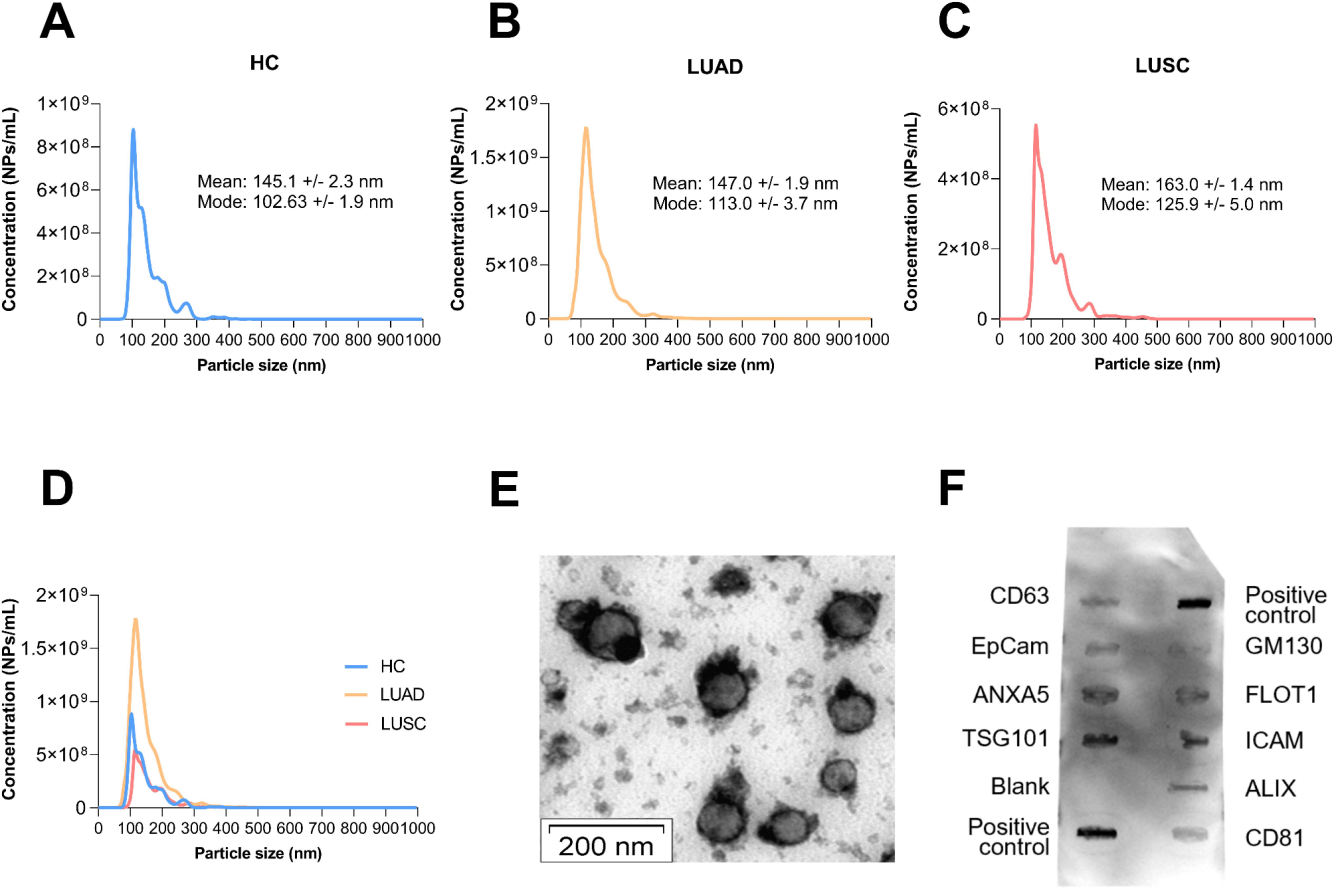
Characterization of serum-derived exosomes (*n* = 5 each sample group). **A**-**C**: Size distribution of exosomes measured by NTA. **D**: Serum exosome concentration (NPs/mL) in HC, LUAD, and LUSC samples as detected with NTA. **E**: Exosomes isolated from NSCLC patients were observed under electron microscopy with 50–150 nm in diameter (bar = 200 nm). **F**: Representative Exo-Check Exosome Antibody Array for detecting exosome markers (CD81, CD63, ALIX, EpCam, ANXA5, and TSG101) and assessing cellular contamination (GM130)

To investigate the amount of WNT5A in vesicle-free and vesicle-bound fractions, WNT5A levels were measured in total serum, exosome-free serum, and serum-derived exosomes. The studied patient cohort was not in therapy at the time of serum sampling (Fig. 1A-B). Healthy volunteers provided the control serum samples (HC). The quality of exosomes was determined by the morphology and size of exosomes, and the detection of exosome-specific markers (Fig. 2A-F). NTA revealed not only that the exosome quality was suitable for further investigation but also that the circulating exosome number was greater in LUAD patients than in either HCs (1.6-fold) or in LUSC patients (2.2-fold). Whereas the exosome concentration in LUSC patients was not just lower than in LUAD patients but even lower than in HCs (0.72-fold).

### WNT5A in blood serum and serum exosomes

**Fig. 3:**
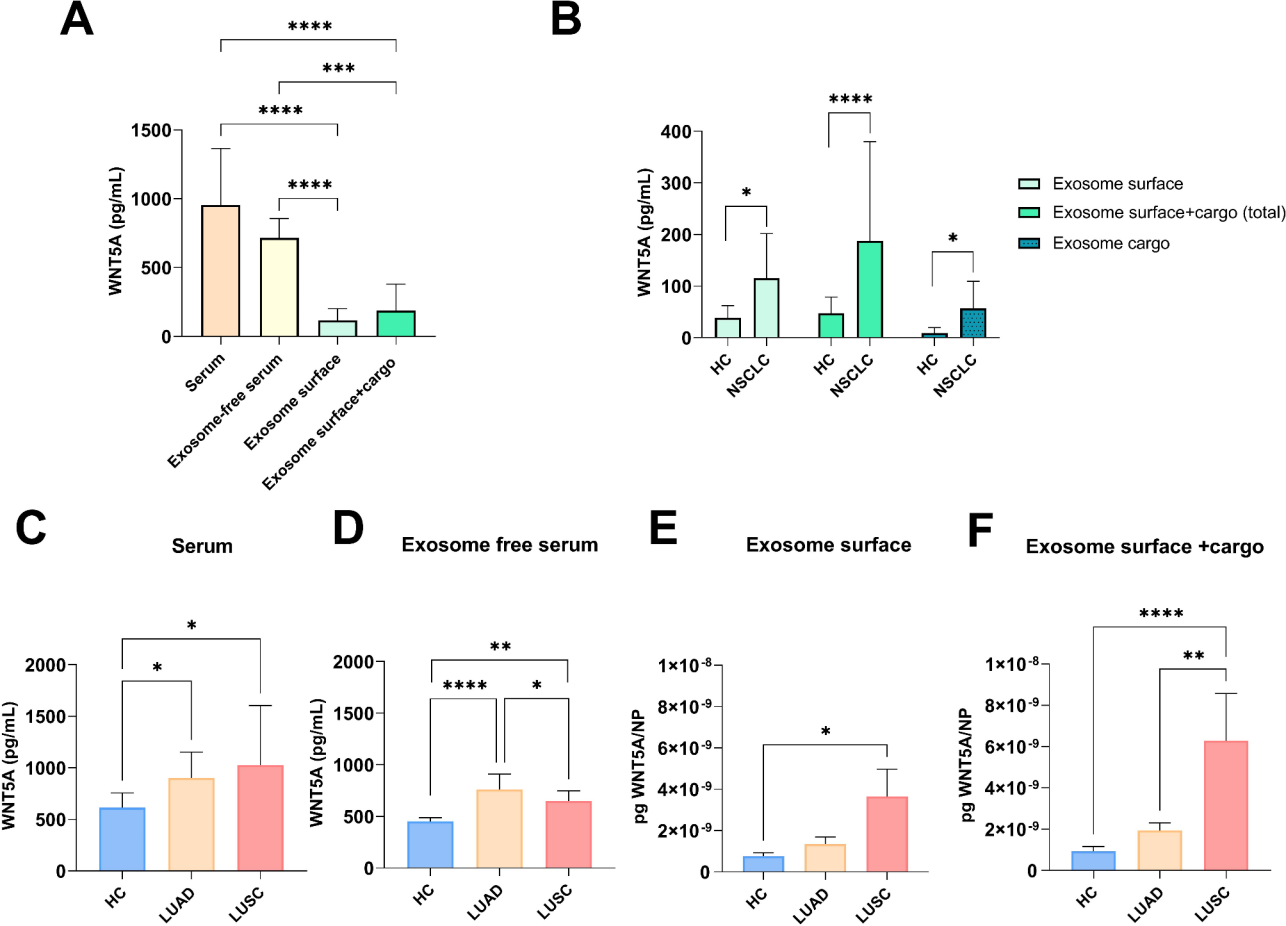
WNT5A levels in various fractions of the serum. **A**: The concentration of WNT5A protein in serum, supernatant, and exosomes. **B**: Distribution of WNT5A within exosomes in NSCLC and HC samples. Quantitative analysis of WNT5A levels in serum (**C**) and supernatant (**D**) among HC, LUAD and LUSC patients. **E**: WNT5A levels pg/particle as exosome surface **F**: WNT5A levels pg/particle as exosome surface + cargo

Figure 3 displays the amount of WNT5A in total serum, exosome-free fraction of the serum, and exosomes both on the exosome surface and in the exosome lumen as cargo. Statistical significance was not detected between total serum WNT5A and exosome-depleted serum WNT5A (Fig. 3A). However, it revealed that significantly less WNT5A protein is transported in exosomes than in the exosome-free fraction (*p* ≤ 0.0001)(Fig. 3A). To further examine the distribution and proportion of WNT5A in exosomes, the isolated exosomes were resuspended in RIPA buffer to disrupt the exosome membrane and to measure WNT5A levels as a combination of surface and lumen (cargo) WNT5A (Fig. 3A). When the exosome lumen and the exosome surface were measured together, a higher level of WNT5A protein was detected, although the increase was not statistically significant (Fig. 3A).

Elevated WNT5A levels were detected in NSCLC patients compared to HCs in all examined serum fractions (Fig. 3B). However, when the total serum and exosome-free serum of LUAD and LUSC patients were analysed separately, a significant difference between the two subtypes was detected only in the exosome-free serum fraction (Fig. 3C and D), where LUSC samples contained less soluble WNT5A compared to LUAD (*p* = 0.051) (Fig. 3D).

Considering that LUAD patients’ sera had significantly higher particle numbers than LUSC patients (Fig. 2), WNT5A levels were also calculated as pg/particle to measure the level of WNT5A/exosomes. The result revealed that LUSC samples carry significantly greater amounts of WNT5A on the exosome surface (Fig. 3E) than either HCs or LUAD exosomes do. Similarly for exosome-free serum fraction, we found a significant difference between the total WNT5A exosome content of patients with LUAD and LUSC (exosome surface + cargo, Fig. 3F). WNT5A content/particle in LUAD and HC exosomes did not differ significantly (Fig. 3E-F).

### WNT5A as a biological marker

To ensure the results are applicable for clinical use, we determined the optimal cut-off values of serum, exosome-free serum, normalized surface exosome, and normalized surface + cargo exosome WNT5A levels.

The cut-off value of WNT5A in the exosome-free serum was 740 pg/mL. Cut-off values were also calculated for WNT5A/particle number. On exosome surface, 1.8 × 10^− 9^ pg of WNT5A/NP was determined as cut-off value, while 2.5 × 10^− 9^ pg WNT5A/NP for exosome surface + cargo.

**Fig. 4:**
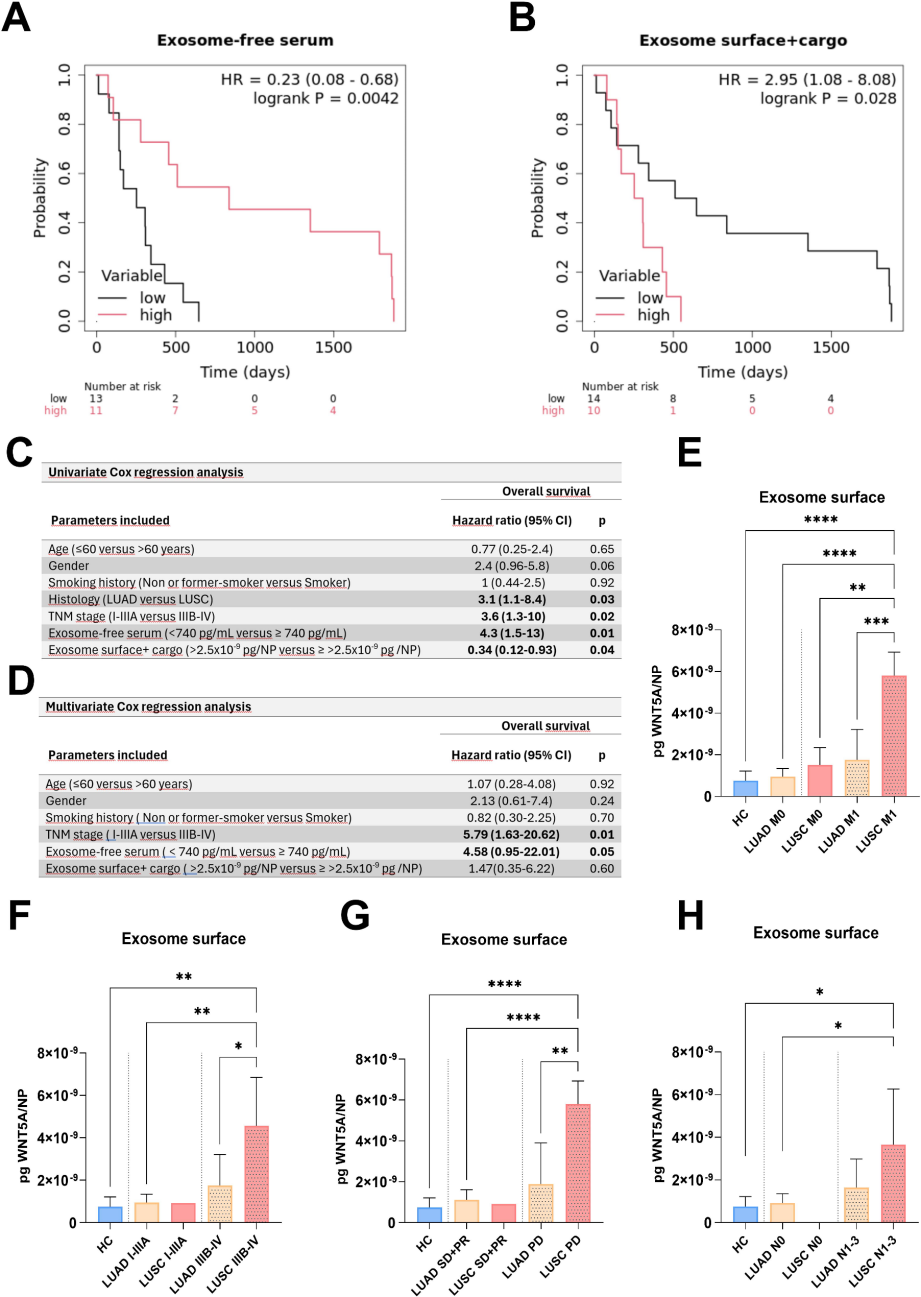
WNT5A levels associated with overall survival, disease stage and therapy response. **A**: Kaplan–Meier O/S distributions stratified by supernatant WNT5A level (cut-off: 740 pg/mL) (*p* = 0.0042, from log-rank test). **B**: Kaplan–Meier O/S distributions stratified by exosome surface + cargo WNT5A level (cut-off: 2.5 × 10^− 9^ pg WNT5A/ NP) (*p* = 0.028, from log-rank test). WNT5A on the surface of exosomes is elevated in the LUSC subtype and associated with distant metastasis (**C**), Univariate Cox regression analysis (D) Multivariate Cox regression analysis (**E**), positive lymph node status (**F**) and lack of response to therapy (**G**). **D**: ROC analysis was performed to calculate sensitivities and specificities for differentiation between LUAD and LUSC metastatic patients. AUC represents the diagnostic capacity. **H**: WNT5A transported on the exosome surface is a suitable marker in distinguishing LUAD patients with PD from LUSC patients with a lack of therapy response. (PR, partial response; PD, progressive disease; SD, stable disease)

In patients with low levels of WNT5A in exosome-free serum (< 740 pg/mL), the OS was shorter compared to those with higher WNT5A levels (≥ 740 pg/mL) (logrank *p* = 0.0042; HR = 0.23; 95% CI 0.08–0.68; Fig. 4A). Conversely, patients with low exosome surface + cargo WNT5A levels (< 2.5 × 10^− 9^ pg WNT5A/NP) had a longer O/S compared to those with high WNT5A levels (≥ 2.5 × 10^− 9^ pg WNT5A/NP) in their exosomes (logrank *p* = 0.028, HR = 2.95; 95% CI 1.08–8.08; Fig. 4B). In addition, univariate analysis revealed that histology (*p* = 0.03, HR = 3.1; 95% CI 1.1–8.4) and TNM stage (*p* = 0.02, HR = 3.6; 95% CI 1.3–10) **were** also a predictor of O/S. However, by multivariate analysis, only exosome-free serum (*p* = 0.05, HR = 4.58; 95% CI 0.95–22.01) and TNM stage (*p* = 0.01, HR = 5.79; 95% CI 1.63–20.62) were found as an independent predictor for O/S. (Fig. 4C/D).

Furthermore, elevated WNT5A/particle on the exosome surface at the therapy baseline was associated with distant metastasis in patients with LUSC (*p* = 0.0056, Fig. 4D). Consequently, more advanced stages of the disease (*p* = 0.0118) (Fig. 4E) and lymph node involvement (*p* = 0.0443) (Fig. 4F) were significantly associated with elevated levels of WNT5A on particle surfaces (> 1.8 × 10^− 9^ pg of WNT5A/NP). Additionally, a high level of WNT5A on the exosome surface/particle (> 1.8 × 10^− 9^ pg of WNT5A/NP) can also predict a lack of response to therapy in LUSC patients (*p* = 0.0073) (Fig. 4G).

In contrast, no such correlations were detected in LUAD patients in any of the examined parameters, indicating that elevated WNT5A on the surface of exosomes only plays a significant role in the LUSC subtype. Supplementary Table 2 demonstrates the ROC curve evaluation of the diagnostic efficacy of different fractions of extracellular WNT5A. The exosome-free serum WNT5A yielded the highest AUC value:0.9702, [CI]: 0.9099 to 1.000) comparing NSCLC patients with the HC group, separating cancer patients from healthy donors. The second highest AUC value was calculated for WNT5A in exosome surface + cargo, which the most accurately distinguished LUAD and LUSC subtypes (AUC: 0.9286, [CI]: 0.8194 to 1.000, Supplementary Table 2).

An independent validation set would be required to confirm the reliability and generalizability of the cut-off values for clinical use at later stages of biomarker development.

## Discussion

WNT5A has a consistent association with various cancer types [30], yet its potential as a biomarker remained largely unverified. In the present manuscript, we postulated that separating vesicle-free and vesicle-bound fractions of the serum might have reliably different WNT5A levels and yield predictive, prognostic, or diagnostic value for NSCLC.

We initially observed that elevated levels of WNT5A in resected tumour tissue were not associated with diminished OS and did not differentiate NSCLC subtypes [2]. However, significant differences were detected in WNT5A levels between the LUAD and LUSC subtypes by separating the exosome-free serum and various exosome fractions. LUSC patients had lower levels of circulating exosomes compared to LUAD patients or HCs. Moreover, LUSC exosomes contained significantly more WNT5A both on their surface and inside as cargo, in comparison to LUAD patients. Additionally, the exosome-free serum of LUAD patients had notably higher levels of WNT5A than LUSC patients, indicating that the two subtypes of NSCLC have distinct extracellular WNT5A profiles suggesting that the location of WNT5A may play a significant role in the clinical outcome.

**Fig. 5:**
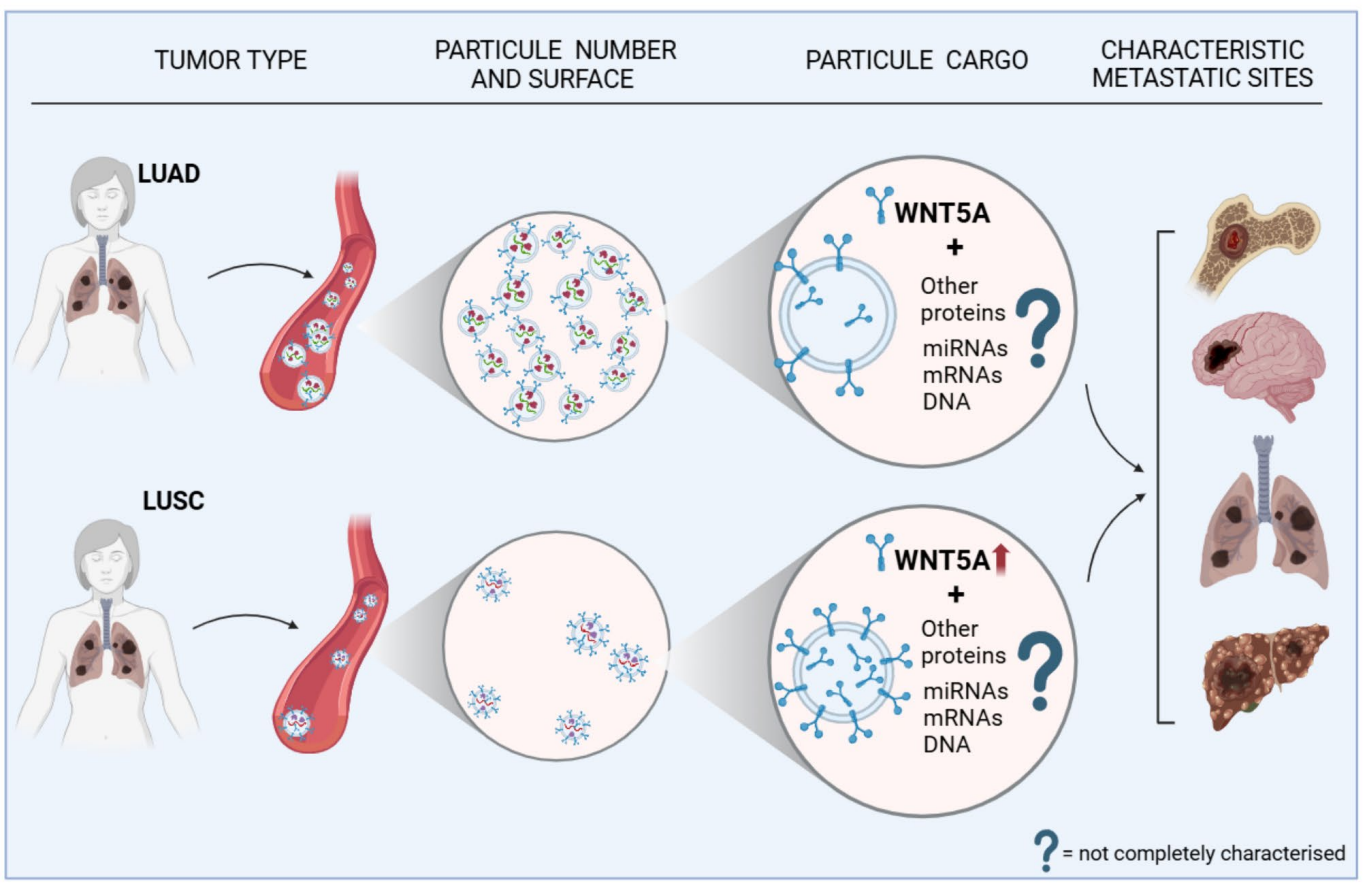
Schematic summary diagram of WNT5A exosome characteristics in sera of LUAD and LUSC patients

Previous research into the role of vesicle-free and vesicle-bound forms of WNT5A has shown that the ratio of secreted versus vesicle-bound WNT5A depends on the cell type and the cellular context [31, 32]. While secreted WNT5A appears to promote a more aggressive cancer phenotype, WNT5A in extracellular vesicles may exhibit context-dependent effects, potentially inhibiting or promoting cancer progression [33, 34]. In our study, high levels of WNT5A bound to exosomes were associated with LUSC patients and shorter OS, while higher WNT5A in exosome-free serum predicted better survival associated with LUAD, suggesting that the context of WNT5A presentation and mode of secretion is crucial for its function in cancer [35, 36]. In exploring the reasons for reduced overall survival in LUSC, we reviewed the literature and found that not only do the exosome numbers and WNT5A levels differ (Fig. 5), but various other cargo molecules do as well. While LUSC exosomes also contain TP63 and KRT5 (Keratin5) mRNA, LUAD exosomes carry CEACAM6 (carcinoembryonic antigen cell adhesion molecule 6) and SFTB (surfactant protein B) mRNAs [37]. TP63 is characteristic in squamous cell carcinomas [38] and, if TP63 protein is combined with SOX2 it is known to promote LUSC progression and serve as super-enhancers in squamous tumors [39]. Our WNT5A data, along with existing literature, were analyzed using Ingenuity Pathway Analysis (IPA, Qiagen), revealing the complexity of the affected signaling pathways (Supplementary Fig. 2). This analysis indicated a significantly higher complexity in LUSC. We also theorized that the membrane protein WNT5A can facilitate the delivery of exosomes to target sites in both subtypes of NSCLC. This is particularly relevant because the common metastatic sites for both subtypes including the lung, bone, liver, and brain tissue, have receptors for WNT5A, such as ROR2 and FZDs [40, 41].

**Fig. 6:**
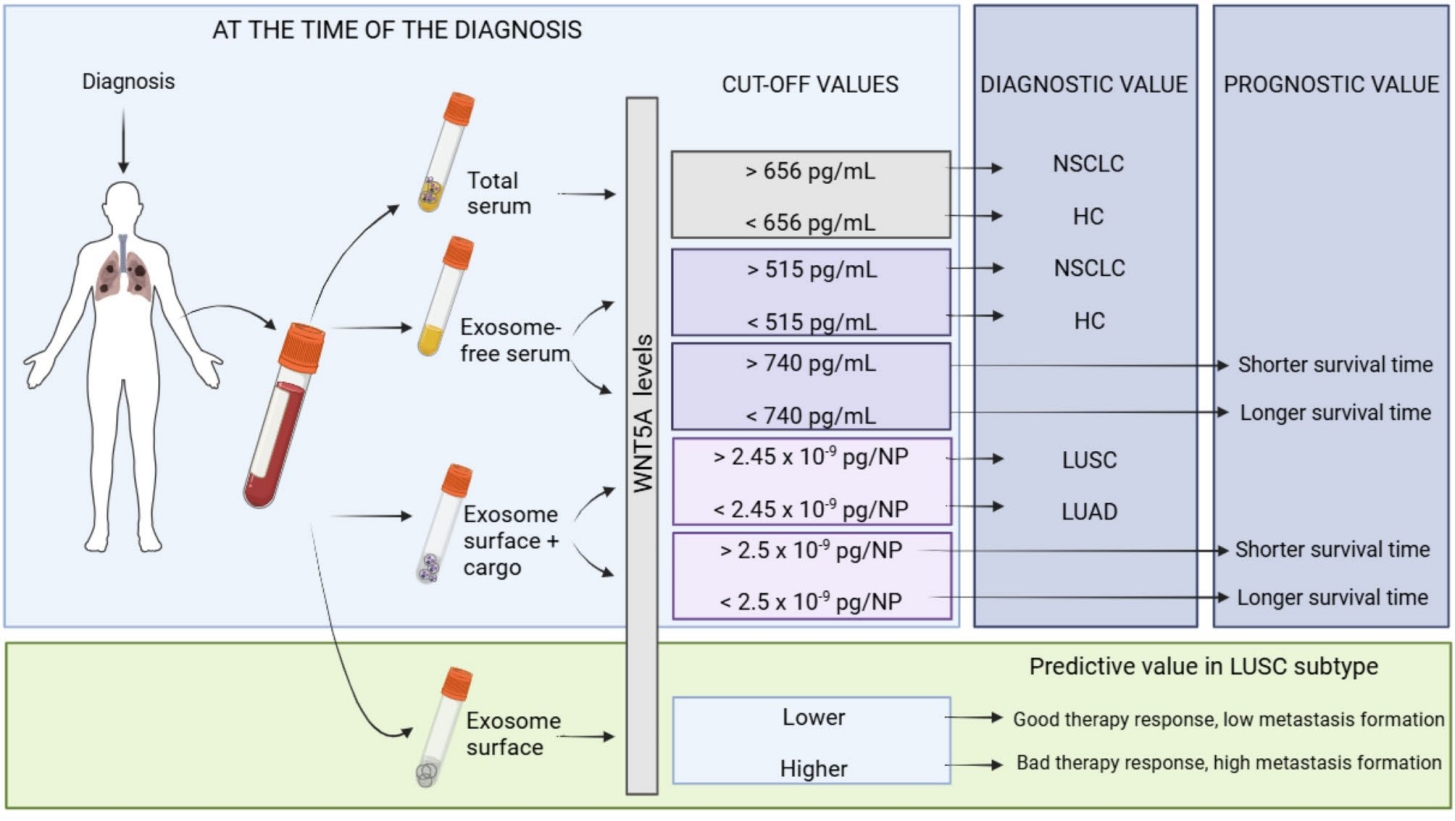
Schematic diagram of the predictive, prognostic and diagnostic role of peripheral WNT5A in LUAD and LUSC patients. The cut-off values are summarized for each application. (Further validation is needed to confirm that the cut-off values are not data-set dependent)

Further studies are certainly needed to elucidate the underlying selection and sorting process of WNT5A into extracellular space and vesicles to elucidate the distinctively different effects on survival. Understanding such differences might also lead to the identification of novel therapeutic targets, which are especially important since exosome-associated high WNT5A level is an indicator of aggressive disease with higher metastatic potential and reduced OS [42]. Significantly, a simple blood sample and measuring WNT5A in serum and exosome fractions at the time of diagnosis can identify advanced disease stage, lymph node involvement, distant metastasis, predict lack of therapy response, and reduced OS in LUSC. In LUAD the exosome-associated WNT5A levels were significantly lower and associated with higher OS time. The difference in location of serum WNT5A is an additional diagnostic biomarker which differentiates LUSC from LUAD. While other studies found that high WNT5A expression is somehow associated with poor prognosis in NSCLC patients [43], our study is the first one to identify the precise location and diagnostic and prognostic value of serum and serum-derived exosome-associated WNT5A with specified cut-off values (Fig. 6). Although smaller sample availability leads to reduced statistical power, the results are promising and warrant further validation in a larger clinical cohort to confirm the clinical utility of the extracellular WNT5A as a biomarker.

In summary, measuring specific extracellular forms of WNT5A in liquid biopsies can provide important clinical information that complements tissue-based biomarkers and supports personalised clinical decision-making. The current data has significant limitations and represents only the first step in biomarker discovery. Independent validation is essential but challenging at this time due to the absence of serum exosome protein databases. Further studies and statistical power analyses are needed to confirm our findings.

## Electronic supplementary material

Below is the link to the electronic supplementary material.

Supplementary Material 1

## Data Availability

The data that support the findings of this study are available from the authors upon reasonable request.

## Declarations

### Clinical trial number

N/A.

## List of Abbreviations

LC: Lung cancer
NSCLC: Non-small cell lung cancer
LUAD: Lung Adenocarcinoma
LUSC: Lung Squamous Cell Carcinoma
TKI: Tyrosine kinase inhibitor
EGFR: Epidermal growth factor receptor
KRAS: Kirsten rat sarcoma viral oncogene homologue
EML4–ALK: echinoderm microtubule-associated protein-like 4–anaplastic lymphoma kinase
BRAF: B-Raf proto-oncogene serine/threonine kinase
MET: hepatocyte growth factor receptor tyrosine kinase
ROS1: ROS proto-oncogene 1
RET: rearranged during transfection proto-oncogene
NTRK: neurotrophic tyrosine receptor kinase
HER2: human epidermal growth factor receptor 2
FGFR 1–3: fibroblast growth factor receptors 1–3
PDGFRA: platelet-derived growth factor receptor alpha
PTEN: phosphatase and tensin homologue
TP53: tumour protein p53
EPHA2: ephrin type A receptor 2
AKT1: alpha serine/threonine-protein kinase
PD: progressive disease
PR: partial response
SD: stable disease
