# Supplementary material for: Serum and exosome WNT5A levels as biomarkers in non-small cell lung cancer
Source: Respir Res. 2025 Apr 13;26:141. doi: 10.1186/s12931-025-03216-7 (PMC11995597; doi:10.1186/s12931-025-03216-7)
Supplement: Supplementary file 1 — Supplementary Material 1 [file 12931_2025_3216_MOESM1_ESM.docx]

**Supplementary Material**

**Serum and Exosome WNT5A Levels as Biomarkers in Non-Small Cell Lung Cancer**

Torok^1,2^, Zsofia; Garai^1^, Kitti; Bóvári-Biri^1^, Judit; Adam^1^, Zoltan; Miskei^1^, Judith A; Kajtar^3^, B; Sarosi^2^, Veronika; Pongracz^1^, Judit E

^1^Department of Pharmaceutical Biotechnology, Faculty of Pharmacy, University of Pecs, 2 Rokus Str, Pecs, H-7624, Hungary

^2^Department of Pulmonology, 1st Internal Medicine; The Medical School and Clinical Centre, University of Pecs, 12 Szigeti Str, Pecs, H-7624, Hungary

^3^Department of Pathology, The Medical School and Clinical Centre, University of Pecs, 12 Szigeti Str, Pecs, H-7624, Hungary

[torok.zsofia@pte.hu](mailto:torok.zsofia@pte.hu); [garai.kitti@pte.hu](mailto:garai.kitti@pte.hu); [bovari.judit@pte.hu](mailto:bovari.judit@pte.hu); [adam.zoltan.mihaly@pte.hu](mailto:adam.zoltan.mihaly@pte.hu); [miskei.judith@pte.hu](mailto:miskei.judith@pte.hu); [kajtar.bela@pte.hu](mailto:kajtar.bela@pte.hu); [sarosi.veronika@pte.hu](mailto:sarosi.veronika@pte.hu); [pongracz.e.judit@pte.hu](mailto:pongracz.e.judit@pte.hu)

Running title: Exosome WNT5A as a predictive biomarker in NSCLC

"The authors declare no potential conflicts of interest."

**Correspondence:**

Prof Dr Judit E Pongracz

Department of Pharmaceutical Biotechnology,

Faculty of Pharmacy,

University of Pecs,

2 Rokus Str,

Pecs, H-7624, Hungary

[pongracz.e.judit@pte.hu](mailto:pongracz.e.judit@pte.hu);

Tel. (work): +36 72 536 000/29250,

Tel. (mobile): +36 30 435 7944

| TOTAL | LUAD  n=14 | LUSC  n=10 | p |
| --- | --- | --- | --- |
| Mean age | 69 | 67 |  |
| Gender |  |  | 0.4212^A^ |
| Male | 7 | 7 |  |
| Female | 7 | 3 |  |
| Smoking history |  |  | 0.3519^B^ |
| Non-smoker | 2 | 0 |  |
| Former- smoker | 7 | 5 |  |
| Smoker | 4 | 5 |  |
| TNM stage |  |  | 0.9999^A^ |
| I-IIIA | 5 | 3 |  |
| IIIB-IV | 9 | 7 |  |
| Tumor invasion |  |  | 0.4015^A^ |
| T_1_-T_2_ | 7 | 3 |  |
| T_3_-T_4_ | 6 | 7 |  |
| Lymphatic invasion |  |  | 0.3394^A^ |
| No | 4 | 1 |  |
| N_1_-N_3_ | 9 | 9 |  |
| Distant metastasis |  |  | 0.9999^A^ |
| M_0_ | 6 | 4 |  |
| M_1_ | 8 | 6 |  |

**Supplementary Table 1**. Patient characteristics at pre-treatment baseline. Statistical analyses were performed using Fisher’s exact (^A^) and chi-square (^B^) tests.

**
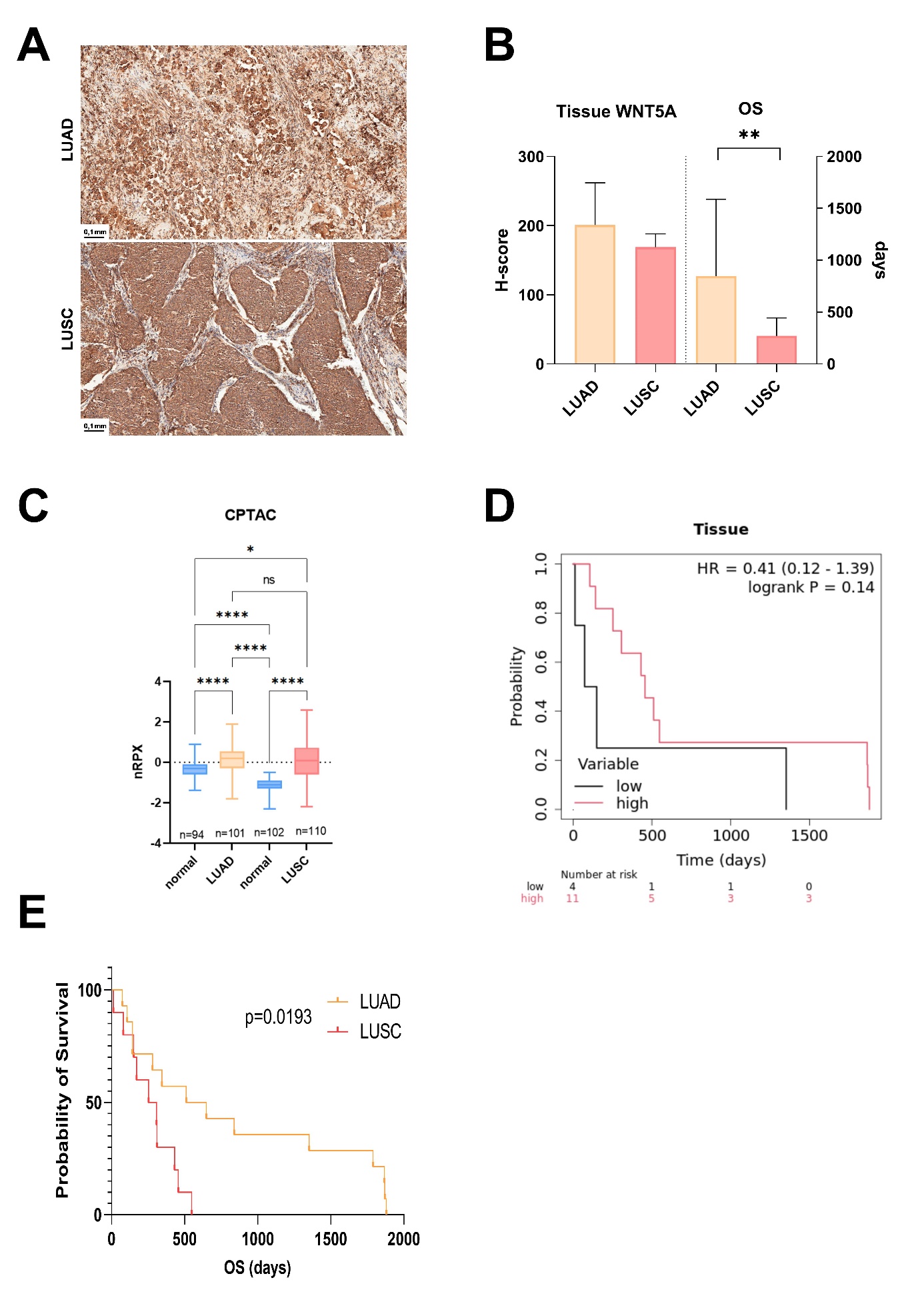
**

**Supplementary Figure 1.**

**WNT5A protein in NSCLC solid tumor tissues and overall survival (O/S)**. Representative images of WNT5A protein expression detected with immunohistochemical staining in LUAD (**A**) and LUSC (**B**) specimens (magnification 10X); (**C**) Independent dataset analysis (**D**) WNT5A was scored via a semiquantitative method based on the H-score; and (**E**) O/S of NSCLC patients.

**SupplementaryTable 2.** Cut-off values of WNT5A are indicated by ROC curve analysis in each group.

|  | **WNT5A** | | | | | |
| --- | --- | --- | --- | --- | --- | --- |
|  | **Serum** | | **Exosome-free serum** | | | |
|  | **NSCLC** | **HC** | **NSCLC** | **HC** | **LUAD** | **LUSC** |
| **Boundary line (pg/mL)** | >656 | <656 | >515 | <515 | > 737 | 515 >x<737 |
| **AUC (95% CI)** | 0.869 (0.6728 to 1.000) | | 0.9702 (0.9099 to 1.000) | | 0.7714 (0.5667 to 0.9762) | |
| **Sensitivity %** | 91.67 | | 95.83 | | 90 | |
| **Specificity %** | 85.71 | | 100 | | 71.43 | |
| **P-value** | 0.0034 | | 0.0002 | | 0.0261 | |
|  | **Exosome surface** | | **Exosome surface+cargo** | | | |
|  | **NSCLC** | **HC** | **NSCLC** | **HC** | **LUAD** | **LUSC** |
| **Boundary line (pg/NP)** | >9.1x10^-10^ | <9.1x10^-10^ | >1.35x10^-9^ | <1.35x10^-9^ | <2.45x10^-9^ | >2.45x10-9 |
| **AUC (95% CI)** | 0.7857 (0.5760 to 0.9954) | | 0.9018 (0.7708 to 1.000) | | 0.9286 (0.8194 to 1.000) | |
| **Sensitivity %** | 71.43 | | 83.33 | | 90 | |
| **Specificity %** | 85.71 | | 85.71 | | 92.86 | |
| **P-value** | 0.00367 | | 0.0014 | | 0.0004 | |

**
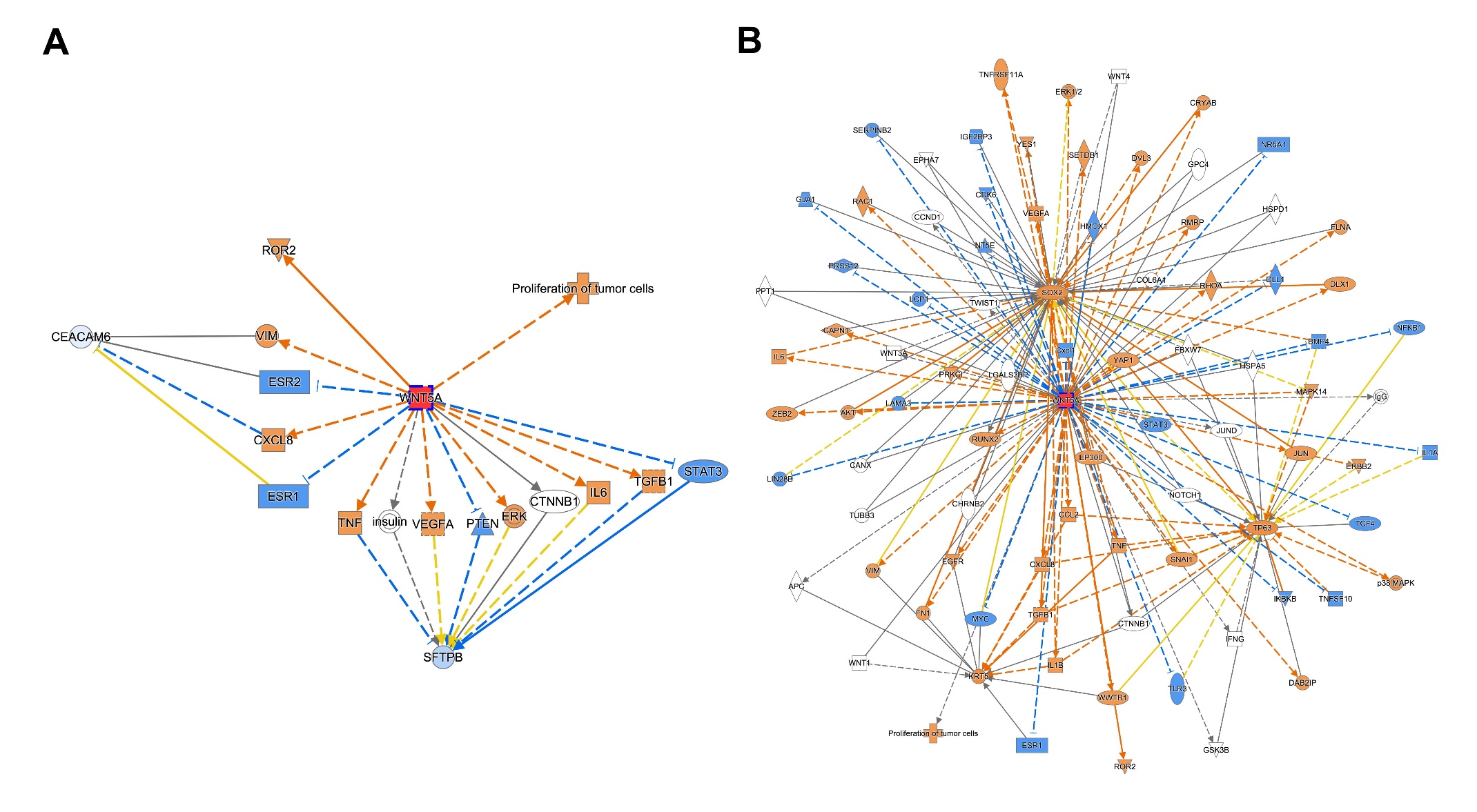
**

**Supplementary Figure 2.** IPA analysis of LUAD **(A)** and LUSC **(B)** exosome protein cargo demonstrating LUSC pathways complexity**.**
